# *GATA3* as a Blood-Based RNA Biomarker for Idiopathic Parkinson’s Disease

**DOI:** 10.3390/ijms241210040

**Published:** 2023-06-12

**Authors:** Shubhra Acharya, Andrew I. Lumley, Lu Zhang, Mélanie Vausort, Yvan Devaux, on behalf of the NCER-PD Consortium

**Affiliations:** 1 Cardiovascular Research Unit, Department of Precision Health, Luxembourg Institute of Health, L-1445 Strassen, Luxembourg; shubhra.acharya@lih.lu (S.A.); andrew.lumley@lih.lu (A.I.L.); melanie.vausort@lih.lu (M.V.); 2 Faculty of Science, Technology and Medicine, University of Luxembourg, L-4365 Esch-sur-Alzette, Luxembourg; 3 Bioinformatics Platform, Luxembourg Institute of Health, L-1445 Strassen, Luxembourg; lu.zhang@lih.lu

**Keywords:** blood-based biomarker, Parkinson’s disease (PD), diagnosis, *GATA3*

## Abstract

Finding novel biomarkers for Parkinson’s disease (PD) is crucial for early disease diagnosis, severity assessment and identifying novel disease-modifying drug targets. Our study aimed at investigating the *GATA3* mRNA levels in whole blood samples of idiopathic PD (iPD) patients with different disease severities as a biomarker for iPD. The present study is a cross-sectional, case-control study, with samples obtained from the Luxembourg Parkinson’s cohort (LuxPARK). iPD (N = 319) patients, along with age-matched controls without PD (non-PD; N = 319) were included in this study. Blood *GATA3* mRNA expression was measured using quantitative reverse transcription PCR (RT-qPCR) assays. The capacity of *GATA3* expression levels to establish the diagnosis of iPD (primary end-point) and assess disease severity (secondary end-point) was determined. The blood levels of *GATA3* were significantly lower in iPD patients, compared to non-PD controls (*p* ≤ 0.001). Logistic regression models showed a significant association of *GATA3* expression with iPD diagnosis after adjustment for the confounders (*p* = 0.005). Moreover, the addition of *GATA3* expression to a baseline clinical model improved its iPD diagnosis capacity (*p* = 0.005). There was a significant association of *GATA3* expression levels with the overall disease severity (*p* = 0.002), non-motor experiences of daily living (nm-EDL; *p* = 0.003) and sleep disturbances (*p* = 0.01). Our results suggest that *GATA3* expression measured in blood may serve as a novel biomarker and may help in the diagnosis of iPD and assessment of disease severity.

## 1. Introduction

Parkinson’s disease (PD) is the second most common neurodegenerative disease after Alzheimer’s disease. Multiple alterations in the neurological pathways leading to the accumulation of aberrant forms of α-synuclein (α-syn), decreased mitochondrial function, decreased dopamine production and the death of dopaminergic neurons are the root causes of PD [1]. The disease initiates with the appearance of non-motor symptoms that gradually progress to motor symptoms, significantly compromising patients’ quality of life [2]. PD affects more than 10 million people worldwide, with more than 1% of the population over 65 suffering from the disease. Alarmingly, the number of PD sufferers is predicted to double by 2030, predominantly because of ageing populations and the continued use of toxic pesticides in many agricultural processes [3]. The current treatment for PD remains symptomatic. With the absence of disease-modifying therapies for PD, there is also a need for improved diagnosis and stratification strategies. The diagnosis of PD is primarily based on the patients’ clinical symptoms. Thus, in the management of PD, reliable biomarkers for early diagnosis and for risk stratification are urgently needed, and could help to drive personalized medicine.

Recent studies have shown the involvement of inflammation and peripheral immunity in the development of PD and its potential in identifying novel biomarkers and disease-modifying targets [4,5]. Indeed, T-cell infiltration has been observed in the brain of PD patients and in in vivo PD models [6,7]. The presence of CD4+ and CD8+ T-cells in the brain has been linked to neuroinflammation, synucleinopathies and progressive dopaminergic degeneration [6,8]. In the blood, CD8+ T-cells are significantly reduced in PD patients when compared to healthy controls, and this is associated with disease severity [9]. In addition, T-cell specific transcription factors were observed to be differentially expressed in CD4+ T lymphocytes of PD patients with motor complications and in early idiopathic rapid eye movement sleep behavior disorder subjects [10,11]. The GATA binding protein 3 (GATA3) is one of many transcription factors expressed by T-cells. It is involved in their development, differentiation and activation, and is vital for regulating the immune response [12]. Additionally, *GATA3* has a role in embryonic development, specifically the development and maintenance of the central nervous system by regulating gene expression in neurons and glia [13]. In PD, *GATA3* was recently shown to be associated with the LRRK2-p.Gly2019Ser variant in early neurodevelopment [14]. Similarly, *GATA3* can also transcriptionally regulate the activity of tyrosine hydroxylase (TH) by increasing the number of TH-positive neurons [15]. TH is an enzyme essential for dopamine production and is important in the pathogenesis of PD [16]. *GATA3* is a master regulator of T helper 2 (Th2) cell development, and plays a role in the nervous system [17,18]; however, its expression in the circulation, and therefore its biomarker potential, has not been studied in the context of PD.

The majority of biomarker-based studies have used patient cerebrospinal fluid (CSF) samples to assess biomarkers associated with the progression and severity of PD [19]. However, the global use of these CSF-based biomarker still remains limited. As the collection of CSF is invasive and needs to be performed by a skilled health worker, finding biomarkers that can be measured in the blood would be quicker, easier, less painful and less hazardous to perform. In this regard, RNAs are modulated in PD and can be measured in patient blood to develop novel diagnostic strategies [20,21]. Taking into account some of the qualities of an ideal biomarker—to be safe, minimally invasive, inexpensive and rapid but accurately measurable—we sought to measure the expression of *GATA3* in the blood of idiopathic PD (iPD) patients and controls without PD (non-PD) using quantitative reverse transcription PCR (RT-qPCR). We additionally studied the relation of *GATA3* expression with disease severity in iPD patients with varying motor and non-motor complications. Finally, we employed parametric models to evaluate the performance of blood *GATA3* expression levels as a potential biomarker for iPD. This might allow us a feasible opportunity of using an easily accessible blood-based biomarker for iPD patients with distinct disease severities and help with patient stratification.

## 2. Results

### 2.1. Clinical Characteristics

The Luxembourg Parkinson’s cohort (LuxPARK) comprised approximately 1700 patients including controls, iPD patients and patients with atypical Parkinsonism [22]. The present study focused on 319 iPD patients and 319 non-PD controls from this cohort. The number of samples used in the present study was based on sample size calculations with an error rate of 5% and power of at least 80%. The demographic and clinical characteristics of the two groups are summarized in Table 1. Patients in the iPD and non-PD groups were of a comparable age. The percentage of female participants (33.2%) in the iPD group was significantly lower than males (66.7%; *p* ≤ 0.001), representing the global sex differences in PD [23,24]. The iPD patients had higher levels of white blood cells and granulocytes, and lower levels of lymphocytes than the non-PD controls.

### 2.2. Evaluation of GATA3 as a Blood-Based Diagnostic Biomarker for iPD

**Figure 1:**
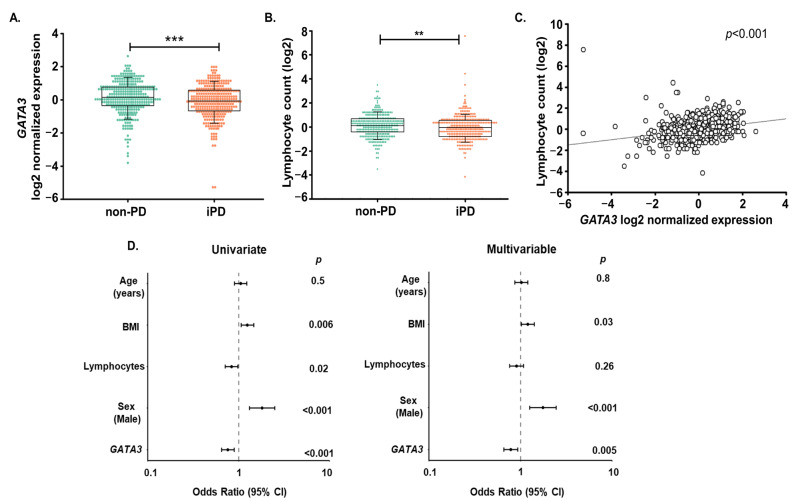
*GATA3* as a diagnostic biomarker for idiopathic Parkinson’s disease (iPD). (**A**) *GATA3* expression was significantly lower in iPD patients (*n* = 319) compared to the non-PD controls (*n* = 319). (**B**) Lymphocyte counts were significantly lower in iPD patients (*n* = 319) compared to the non-PD controls (*n* = 319). (**C**) Lymphocyte counts in all participants (*n* = 638) were positively associated with *GATA3* expression (Spearman correlation coefficient (r) = 0.341, *p* < 0.001). (**D**) Logistic regression models for iPD diagnosis with forest plots showing the odds ratio (OR) with 95% confidence intervals (CI). *GATA3* was associated with iPD diagnosis (univariate), even after adjustment for age, BMI, lymphocyte counts, and sex (multivariable). The expression of *GATA3* was normalized to *18S* and log2 transformed. ** *p* ≤ 0.01, *** *p* ≤ 0.001; Mann–Whitney Rank Sum Test.

The levels of *GATA3* mRNA were measured in the blood of iPD (*n* = 319) and non-PD controls (*n* = 319), using RT-qPCR. The expression of the housekeeping gene *18S* used for normalization was not significantly different for non-PD controls and iPD subjects (*p* = 0.13) (Figure S1A). Normalized expression of *GATA3* was significantly lower in iPD patients compared to non-PD controls (Figure 1A; *p* ≤ 0.001). A similar pattern was also observed between non-PD and iPD for the *GATA3* raw data (*p* ≤ 0.001; Figure S1B), attesting an absence of bias due to the qPCR normalization process. As *GATA3* is a T-cell transcription factor, we also studied the lymphocyte counts in iPD and non-PD samples. We observed a significant decrease in the lymphocyte counts in iPD patients compared to the non-PD controls (Figure 1B; *p* = 0.017), which was positively associated with *GATA3* expression (Figure 1C; *p* ≤ 0.001). Thus, lymphocyte counts were added as one of the confounders in the statistical measures further described.

To examine the potential of *GATA3* to diagnose iPD, we conducted univariate and multivariable logistic regression analysis. Age, sex, BMI and lymphocyte counts were included as parameters in the baseline model. We observed that *GATA3* expression was associated with iPD diagnosis in the univariate analysis (Odds ratio (OR) = 0.76, 95% Confidence intervals (CI) = 0.64–0.89, *p* ≤ 0.001; Figure 1D). This association remained significant after adjustment with the baseline parameters in the multivariable logistic regression model (OR = 0.78, 95% CI = 0.65–0.93, *p* = 0.005; Figure 1D). The incremental value of *GATA3* to diagnose iPD was examined using the Akaike Information Criterion (AIC). AIC is used to estimate how well the model fits the data it was generated from. The lower the AIC value, the better the model fits. In our results, a decrease in AIC was observed upon addition of *GATA3* (AIC = 864.8) in the baseline model (AIC = 870.5). A significant Likelihood Ratio Test (LRT; *p* = 0.005) when comparing the two models, denoted that adding *GATA3* in the baseline model improves iPD diagnosis (Table 2). Moreover, the addition of *GATA3* was also able to reclassify patients misclassified by the baseline model, as assessed by the Integrated Discrimination Index (IDI = 0.011, *p* = 0.006; Table 2).

### 2.3. Association between GATA3 and Disease Severity

**Figure 2:**
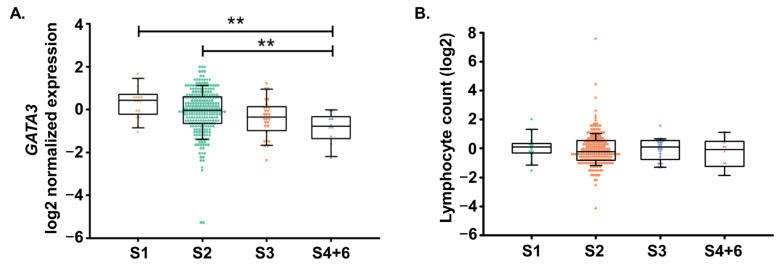
*GATA3* expression in idiopathic Parkinson’s disease (iPD) patients with variable disease severity. (**A**) A decrease in *GATA3* expression levels was observed with increasing disease severity, as assessed by the Hoehn and Yahr Stage (H-Y stage). (**B**) The lymphocyte count was not associated with disease severity. S1 (*n* = 17)—Stage 1 of H-Y Stage, S2 (*n* = 264)—Stage 2 of H-Y Stage, S3 (*n* = 28)—Stage 3 of H-Y Stage, S4 + 6 (*n* = 9 + 1)—Stage 4 and 6 of H-Y Stage. The expression of *GATA3* was normalized to *18S* and log2 transformed. ** *p* ≤ 0.01; One-Way ANOVA Kruskal–Wallis test.

We next studied the association between blood *GATA3* expression levels and disease severity assessed by the Hoehn and Yahr (H-Y) Stages. Here, we observed that blood *GATA3* expression levels were significantly lower in iPD patients with a higher H-Y stage (Figure 2A). Lymphocyte counts were not associated with the H-Y Stage (Figure 2B).

Furthermore, univariate linear regression analysis highlighted the fact that the expression of *GATA3* was significantly negatively associated with disease severity assessed by the H-Y stage (coefficient [95% CI] = −0.108 [−0.16 to −0.04], *p* ≤ 0.001). This association remained significant after the simultaneous inclusion of confounding variables in the multivariable models (coefficient [95% CI] = −0.098 [−0.16 to −0.03], *p* ≤ 0.001); Table 3).

To evaluate the association between *GATA3* and the development of various symptoms of iPD, we performed a linear regression analysis between *GATA3* and the clinical variables in iPD patients (Table S1). While there were no robust associations found between *GATA3* expression levels and motor symptoms (Unified Parkinson’s Disease Rating Scale; UPDRS Part III) or cognitive impairment (Montreal Cognitive Assessment test; MoCA), we observed that *GATA3* was negatively associated with the non-motor aspects of experiences of daily living (nM-EDL) as assessed by the UPDRS Part I score (coefficient [95% CI] = −0.117 [−0.21 to −0.02], *p* = 0.01). This association remained significant after the inclusion of confounders in the multivariable model (coefficient [95% CI] = −0.111 [−0.18 to −0.03, *p* value = 0.003; Table 3). Furthermore, *GATA3* was positively associated with sleep disturbances measured by the Parkinson’s Disease Sleep Scale (PDSS) in the multivariable linear regression analysis (coefficient [95% CI] = 0.101 [0.02 to 0.18], *p* value = 0.01; Table 3), suggesting that lower *GATA3* levels are linked to increased sleep disturbances in iPD patients. Furthermore, considering the impact that pharmacological treatments can have on biomarkers, we explored the effect of treatment using the Levodopa equivalent daily dose (LEDD) score on *GATA3* expression levels. In the linear regression analysis, we found no association (coefficient (95% CI) = −0.079 (−0.258 to 0.100), *p* = 0.386)) between the LEDD and *GATA3* expression levels which does not exclude, however, the fact that other pharmacological treatments may affect *GATA3* expression levels.

## 3. Discussion

The diagnosis of PD remains laborious and dependent upon a series of motor and non-motor tests. With an unmet clinical need to identify and develop disease-modifying therapies for PD, there is also a synchronous necessity to develop consistent biomarkers for early diagnosis and severity assessment. In this study, we examined the potential of blood *GATA3* expression levels as a diagnostic and severity marker for iPD patients in the LuxPARK cohort.

*GATA3* is a member of the GATA family of transcription factors that are zinc finger DNA-binding proteins known to have important roles during early vertebrate development. *GATA3* plays important regulatory roles in T-cell development and Th2 differentiation [12]. It has been shown to be involved in the development of several types of carcinomas, triple negative breast cancer, colorectal cancer and many more forms of cancer [25]. Increased expression of *GATA3* is the most widely used biomarker for breast cancer [26]. *GATA3* is also a TH-specific transcription factor that can regulate TH expression in neurons [15]; however, its use as a biomarker for PD has remained unexplored. Interestingly, T-cell levels are decreased in the blood of PD patients, and this decrease has been shown to be associated with disease severity [9,27]. Kustrimovic et al. examined T-cell specific transcription factors in circulating CD4+ T-cells extracted from the whole blood of PD patients and healthy controls. The authors show a significant decrease in naïve CD4+ T-cells and an increased expression of *GATA3* levels in the CD4+ T-cells of the PD patients compared to controls [27]. Similar to the above studies, in our study, we observed a significant decrease in lymphocytes in iPD, patients compared to the non-PD controls. However, the decrease in lymphocyte counts was not associated with the severity of iPD in the LuxPARK cohort. It is important to note that the quantification of T-cells/lymphocytes in the two studies cited above ([9,27]) was performed using different methods, and results must be inferred cautiously. Additionally, as opposed to the observation by Kustrimovic et. al. [27], we found that the *GATA3* expression in whole blood samples of iPD patients was significantly lower than that of the non-PD controls. This opposing observation could be due to the use of different matrices in the two studies. The above-mentioned study aimed at investigating the role of peripheral adaptive immunity and at establishing CD4+ T-cell-specific molecular signatures in PD [28]. On the other hand, the main aim of our study was to discover novel clinically applicable biomarkers for iPD, hence our choice of easily accessible and usable whole blood samples. These samples comprise a heterogeneous mixture of immune cells, as compared to the purified CD4+ T-cells [28]. Biomarkers based on whole blood samples can be easily applied in clinics, as they require less sample processing steps, whilst providing a comprehensive view of the immune systems’ response to disease. RNA-based biomarkers have shown the potential to improve healthcare in other disease conditions [29,30].

To assess the ability of *GATA3* expression to improve the diagnosis of iPD, we used the LRT to compare the AIC of clinical models with and without *GATA3*. As compared to areas under the receiver operating characteristic curves, the use of corrected AIC allows for the penalizing for the number of predictors, to avoid model overfitting. This approach has already been used in other similar circumstances by ourselves [31] and others [32]. The addition of *GATA3* expression data to a baseline model including risk factors such as age, sex, BMI and lymphocyte counts, significantly improved the diagnostic capacity of the model, as seen by a decrease in AIC and significant LRT. IDI also showed significant improvement in the classification of subjects who had been misclassified by the baseline model. These observations suggest that blood *GATA3* expression could potentially be used as a biomarker to help in the diagnosis of patients with iPD.

The discovery of reliable biomarkers to identify different degrees of severity of the disease is crucial for personalized medicine, and is one of the priorities in PD research. This could be important not only to adjust healthcare and medication and predict disease progression, but also to identify novel disease-modifying therapeutic targets [33]. Thus, in addition to the decreased expression of *GATA3* in iPD patients, we found that *GATA3* expression levels were significantly associated with the overall severity of the disease (H-Y stage) after adjusting for the confounding variables. Along with disease severity, we also show that blood *GATA3* was associated with the nM-EDL and especially with sleep disturbances assessed by the PDSS scale. The nM-EDL score changes rapidly over the first ten years of PD development [34]. Moreover, symptoms relating to sleep disturbances such as excessive daytime sleepiness are early signs of PD, and considered as a risk factor for prodromal PD [35,36]. Keeping this in mind, *GATA3* could potentially be useful in identifying iPD in its early stages, as well as identifying patients who are at risk of developing non-motor symptoms. This could consequently allow the tailoring of treatment and management plans to improve patient care.

### Limitations

Even though the results from our study are encouraging, they must be interpreted considering a few limitations. Firstly, it important to acknowledge that since our study aimed at deciphering the biomarker potential of *GATA3* expression levels in iPD, additional experiments with *GATA3* protein measurements could provide insights into its protein levels. While beyond the scope of the present study, future investigations could include measuring *GATA3* protein levels using Western blotting to complement the mRNA findings and provide a more comprehensive understanding of the role of *GATA3* in iPD. Secondly, granting that we evaluated the association of *GATA3* with PD-associated clinical evaluations, our baseline model could still be underpowered, due to the lack of established imaging or fluid-based biomarker measurements. In this regard, it would be meaningful to have data on fluid biomarkers, such as the recently developed α-syn seed amplification assays (SAA) [37]. In a recent study conducted on the LuxPARK cohort, serum α-syn SAA performed on controls (*n* = 20) and PD (*n* = 20) samples showed a high PD diagnostic performance (AUC = 0.86; (95% CI 0.74–0.99)) [38]. Given the larger sample size in our study, which included 638 participants, extending the α-syn SAA to cover all samples would be essential to facilitate robust analysis, reinforcing the added value of *GATA3* on existing biomarkers. Thirdly, we have performed a cross-sectional analysis in the present study. As LuxPARK is also a longitudinal study, further analysis on longitudinal data could provide insights inn the association of *GATA3* with other clinical variables developed over time, as well as its capacity to predict disease trajectories. Lastly, participants in this study were recruited in a single center, and thus the replication of our findings in other independent cohorts is necessary. Despite these drawbacks, our study illustrates the potential of a blood-based RNA to diagnose and risk stratify PD patients, suggesting a potential role for RNA molecules in PD clinics in the future.

## 4. Materials and Methods

### 4.1. Study Participants

The present work is a cross-sectional study with 638 participants. A total of 319 iPD and 319 age-matched non-PD controls with blood samples available for the present study were obtained from the LuxPARK cohort [22]. The inclusion and exclusion criteria for patients and non-PD controls for the LuxPARK study have been described previously [22]. Briefly, patients above the age of 18 years that fulfilled the United Kingdom Parkinson’s Disease Society (UK-PDS) Brain Bank Clinical Diagnostic Criteria were included in the iPD sample group of the study [39]. Non-PD controls were subjects above 18 years of age. Non-PD controls with a neurodegenerative disease or active cancer and pregnant women were excluded from the study. Motor symptoms and disease staging were assessed by the expert neurologists using the ‘UPDRS’ and the ‘H-Y’ scale, respectively. Early non-motor symptoms such as sleep disturbances or autonomic dysfunction were assessed using the PDSS and the Scale for Outcomes in Parkinson’s disease for Autonomic symptoms (SCOPA-AUT), respectively. Advanced non-motor complications such as cognitive loss and dementia were assessed using the MoCA and the Beck Depression Inventory test (BDI). Patient characteristics are mentioned in Table 1. On inclusion in the LuxPARK study, 2.5 mL of venous blood from each participant was collected in PAXgene^TM^ Blood RNA tubes (PreAnalytiX, Cat. #762165; BD Biosciences, Aalst, Belgium) and stored at the Integrated Biobank of Luxembourg (IBBL).

### 4.2. RNA Extraction and RT-qPCR

Total RNA from PAXgene^TM^ Blood RNA tubes was extracted, quantified and stored according to the ISO/IEC 17025:2017 and ISO 9001:2015 accredited methods [40]. A total of 500 ng of total RNA for each sample was obtained from the IBBL and reverse transcribed to cDNA, using SuperScript^TM^ II reverse transcriptase (Invitrogen Cat. #18064014: ThermoFisher Scientific, Merelbeke, Belgium) and random hexamers, following the manufacturer’s instructions. cDNA was diluted 10 times for final use for quantitative PCR (qPCR). qPCR was carried out using the iQ^TM^ SYBR^®^ Green Supermix (Bio-rad Cat. #1708885; Bio-Rad, Temse, Belgium) on a CFX96 real time PCR system (Bio-rad, Temse, Belgium). Samples were randomly distributed in the qPCR plates. Appropriate controls for the evaluation of genomic DNA contamination, along with inter-run calibrators, were included in each qPCR assay. *18S* ribosomal RNA was used as a housekeeping gene to normalize the expression levels of *GATA3*. Normalized expression (2-ΔCq) was evaluated using the CFX Maestro 2.2 software (Bio-rad, Temse, Belgium). qPCR primers were designed using the online software Primer3. Primer sequences (5′ to 3′)—*18S*: Sense- CGGCGACGACCCATTCGAAC, Anti-sense- GAATCGAACCCTGATTCCCCGTC. *GATA3*: Sense- TCATTAAGCCCAAGCGAAGG, Anti-sense -TCCCCATTGGCATTCCTCCT.

### 4.3. Statistical Analyses

Statistical analysis was performed using SigmaPlot version 14.5 (Systat, Palo Alto, CA, USA). The expression values were log2 transformed for graphical representation. The difference in *GATA3* expression between the two groups (non-PD vs. iPD) was evaluated using a two-tailed *t*-test. For data that did not follow normal distribution, the Mann–Whitney Rank Sum test was used. A one-way Analysis of Variance (ANOVA) Kruskal–Wallis test was used for comparisons involving two or more groups (H-Y stages). Furthermore, R version 4.0.3 (R Foundation, Vienna, Austria) was used for logistic regression analysis to assess the association of *GATA3* expression with the patient diagnosis. Parametric tests were employed to determine the AIC (Akaike Information Criterion), to confirm the strength of the model. The LRT (Likelihood Ratio Test) was used to compare the two models (baseline vs. baseline + *GATA3*). IDI (Integrated Discrimination Improvement) was used to evaluate the ability of *GATA3* to reclassify patients misclassified by the baseline clinical model. Linear regression models were used to explore the association of *GATA3* expression with the clinical variables. Missing data were imputed using the missForest, and further rank normalized using the RankNorm R packages. Other R packages such as Hmisc, rms, lmtest, and glmtoolbox were used to perform the analysis. A *p*-value of ≤ 0.05 was considered significant.

## 5. Conclusions

Patients with iPD from the LuxPARK cohort had significantly lower expression levels of *GATA3* in the blood, which was associated with disease severity, nM-EDL and sleep disturbances. Thus, blood *GATA3* expression represents a promising biomarker for the diagnosis of iPD. Further longitudinal studies are needed to validate the biomarker potential, prognostic value and clinical utility of *GATA3*. Our study brings PD biomarker research one step closer to identifying cost effective, minimally invasive, easy-to-use and reliable biomarkers for iPD.

## Figures and Tables

**Table 1:** Demographic and clinical characteristics of the study population.

Characteristics	Non-PD (*n* = 319)	iPD (*n* = 319)	*p* Value
Age (years)	63.72 ± 7.52	64.36 ± 8.61	0.09
M/F (n)	168:151	213:106	≤0.001
BMI	27.5 ± 4.44	28.35 ± 4.38	0.013
Disease duration (years)	NA	9.2 ± 7.7	NA
H-Y stage	NA	Stage 1–17; Stage 2:264; Stage 3–28; Stage 4–9; Stage 6–1	NA
UPDRS part I	4.19 ± 3.9	10.18 ± 6.17	≤0.001
UPDRS part II	0.98 ±1.92	11.02 ± 7.0	≤0.001
UPDRS part III	2.39 ± 3.72	33.90 ± 12.66	≤0.001
UPDRS part IV	NA	2.11 ± 3.59	NA
MoCA	27.93 ± 1.33	26.40 ± 2.65	≤0.001
BDI	4.99 ± 4.73	8.72 ± 6.10	≤0.001
PDSS	124.2 ± 17.67	104.53 ± 24.39	≤0.001
REM	2.10 ± 1.71	4.61 ± 3.18	≤0.001
SCOPA-AUT	7.33 ± 5.15	14.50 ± 7.57	≤0.001
Sniffin’ sticks test	13.31 ± 1.39	7.63 ± 3.33	≤0.001
LEDD	NA	636 ± 378.84	NA
Blood cell counts (10^3^/mm^3^)			
Lymphocytes	1.88 ± 0.67	1.85 ± 1.47	0.01
Monocytes	0.41 ± 0.30	0.42 ± 0.31	0.44
Granulocytes	3.70 ± 1.27	4.26 ± 1.36	≤0.001
WBCs	6.01 ± 1.58	6.54 ± 2.09	≤0.001
RBCs	4.85 ± 0.57	4.74 ± 0.67	0.14

Numbers are represented as mean ± SD. M/F: Male/Female, BMI: Body Mass Index, iPD: idiopathic Parkinson’s disease, H-Y: Hoehn and Yahr, UPDRS: Unified Parkinson’s Disease Rating Scale, MoCA: Montreal Cognitive Assessment Test, BDI: Beck Depression Inventory test, PDSS: Parkinson’s Disease Sleep Scale, REM: Rapid Eye Movement disorder, SCOPA-AUT: Scale for Outcomes in Parkinson’s disease for Autonomic symptoms, LEDD: Levodopa Equivalent Daily Dose, WBCs: White Blood Cells, RBCs: Red Blood Cells, NA: Not applicable.

**Table 2:** Performance of blood *GATA3* expression levels as a biomarker for idiopathic Parkinson’s disease (iPD) diagnosis.

Prediction Model	AIC	Wald Test *p*	LRT *p*	IDI	IDI *p*
Baseline model	870.5	0.0001	-	-	-
Baseline model + *GATA3*	864.8	0.00002	0.005	0.011	0.006

The baseline model included age, sex, BMI and lymphocyte counts. *p*: *p*-value, AIC: Akaike Information Criterion, LRT: Likelihood Ratio Test, IDI: Integrated Discrimination Index.

**Table 3:** Linear regression analysis in idiopathic Parkinson’s disease (iPD) patients.

**A. Linear Regression for ‘Disease Severity’ in Patients with Ipd as** **Assessed by Hoehn and Yahr Stages**				
**Independent** **Variables**	**Univariate**		**Multivariable**	
	**Coefficient (95% CI)**	***p* Value**	**Coefficient (95% CI)**	***p* Value**
Age (years)	0.034(−0.02 to 0.09)	0.22	0.01(−0.04 to 0.06)	0.66
Sex (male)	−0.170(−0.29 to −0.04)	0.008	−0.17(−0.29 to −0.05)	0.005
Disease duration (years)	0.224(0.12 to 0.32)	<0.001	0.208(0.11 to 0.30)	<0.001
BMI	0.043 (−0.01 to 0.10)	0.16	0.046(−0.01 to 0.10)	0.062
Lymphocyte counts	−0.017(−0.07 to 0.04)	0.57	0.010(−0.05 to 0.07)	0.72
*GATA3*	−0.108(−0.16 to −0.04)	<0.001	−0.098(−0.16 to −0.03)	0.002
**B. Linear Regression for ‘Non-Motor Experiences of Daily Living’ in Patients with iPD as Assessed by UPDRS PART I**				
**Independent variables**	**Univariate**		**Multivariable**	
	**Coefficient (95% CI)**	***p* Value**	**Coefficient (95% CI)**	***p* Value**
Age (years)	0.019(−0.06 to 0.10)	0.66	0.118(0.04 to 0.19)	0.001
Sex (male)	−0.163(−0.36 to 0.03)	0.11	−0.176(−0.32 to −0.03)	0.01
Disease duration (years)	0.396(0.24 to 0.54)	<0.001	−0.346(−0.41 to −0.27)	<0.001
BMI	0.066(−0.03 to 0.16)	0.18	0.075(0.003 to 0.14)	0.04
Lymphocyte counts	−0.069(−0.16 to 0.02)	0.14	−0.057(−0.13 to 0.01)	0.13
*GATA3*	−0.117(−0.21 to −0.02)	0.01	−0.111(−0.18 to −0.03)	0.003
**C. Linear Regression for ‘Sleep Disturbances’ in Patients with iPD as** **Assessed by Parkinson’s Disease Sleep Scale (PDSS)**				
**Independent variables**	**Univariate**		**Multivariable**	
	**Coefficient (95% CI)**	***p* Value**	**Coefficient (95% CI)**	***p* Value**
Age (years)	0.002(−0.09 to 0.09)	0.95	−0.097(−0.17 to −0.01)	0.01
Sex (male)	0.088(−0.12 to 0.30)	0.41	0.064(−0.09 to 0.22)	0.41
Disease duration (years)	−0.437(−59 to −0.27)	<0.001	0.255(0.17 to 0.33)	<0.001
BMI	−0.044(−0.14 to 0.06)	0.40	−0.061(−0.13 to 0.01)	0.11
Lymphocyte counts	0.080(−0.01 to 0.18)	0.11	0.040(−0.04 to 0.12)	0.32
*GATA3*	0.136(0.03 to 0.23)	0.009	0.101(0.02 to 0.18)	0.01

Univariate and multivariable linear regression analysis show significant association between *GATA3* expression and (A) disease severity assessed by the H-Y stages, (B) non-motor experiences of daily living (nm-EDL) assessed by UPDRS Part I, and (C) sleep disturbances assessed by Parkinson’s Disease Sleep Scale (PDSS).

## Data Availability

Data used in the preparation of this manuscript were obtained from the National Centre of Excellence in Research on Parkinson’s Disease (NCER-PD). NCER-PD datasets are not publicly available, as they are linked to the Luxembourg Parkinson’s Study and its internal regulations. The NCER-PD Consortium is willing to share its available data. Its access policy was devised based on the study ethics documents, including the informed consent form, as approved by the national ethics committee. Requests to access datasets should be directed to the Data and Sample Access Committee via email: request.ncer-pd@uni.lu.

## Supplementary Materials

The supporting information can be downloaded at: https://www.mdpi.com/article/10.3390/ijms24120040/s1.
