# Supplementary material for: GATA3 as a Blood-Based RNA Biomarker for Idiopathic Parkinson’s Disease
Source: Int J Mol Sci. 2023 Jun 12;24(12):10040. doi: 10.3390/ijms241210040 (PMC10298393; doi:10.3390/ijms241210040)
Supplement: Supplementary file 1 [file ijms-24-10040-s001.zip › ijms-2441305-supplementary.pdf]

Supplementary file-

Table S1. Linear regression analysis in idiopathic Parkinson's disease (iPD) patients to show association between blood *GATA3* expression and PD associated clinical variable.

Clinical variable	Coefficient	95% CI	*p* Value
Lymphocyte count	0.311	0.210 to 0.412	<0.001
Hoehn and Yahr Stages	−0.108	−0.169 to −0.048	<0.001
PDSS	0.153	0.037 to 0.269	0.010
UPDRS Part I	−0.150	−0.274 to −0.026	0.018
SCOPA-AUT	−0.109	−0.230 to 0.012	0.077
UPDRS Part II	−0.142	−0.303 to 0.018	0.082
REM	−0.097	−0.211 to 0.017	0.094
Sniffin' Sticks score	0.117	−0.030 to 0.264	0.119
UPDRS Part III	−0.111	−0.288 to 0.066	0.219
Disease duration	−0.108	−0.287 to 0.071	0.234
BDI	−0.059	−0.176 to 0.057	0.316
MoCA	0.048	−0.053 to 0.150	0.350
LEDD	−0.079	−0.258 to 0.100	0.386
UPDRS Part IV	−0.055	−0.181 to 0.072	0.397

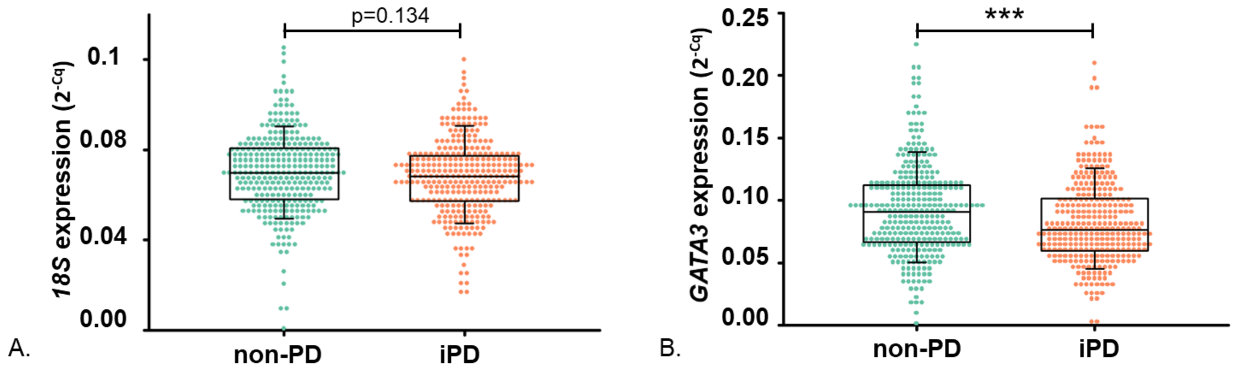

Figure S1. Raw expression levels of 18S and *GATA3*. A) Expression levels of *18S* were comparable between non-PD controls and idiopathic Parkinson's disease (iPD) and B) Expression levels of *GATA3* mRNA were downregulated in iPD compared to non-PD controls. *** $p \leq 0.001$; Mann-Whitney Rank Sum Test.
